# Feeding and Nursing the Sick

**Published:** 1889-06

**Authors:** 


### FEEDING AND NURSING THE SICK.

We extract the following useful hints from a lengthy article which appeared in the Boston *Herald* of May 6:

The dietetic treatment of diseases are as various as are the ills that flesh is heir to. A thorough understanding of them requires even longer and closer study than does that of drugs. In many instances the proper application of foods calls for even greater skill than does correct dosing. The popular idea, doubtless, is that when a person is sick he should be given at certain intervals nutritious and yet easily digested foods. Those two are held to be the prime essentials, and, when met, nurses often feel all is done which can be done.

In nearly all acute diseases nature deprives the patent for a time of all appetite. One would imply from this that at such times it were best to withhold nourishment until there was a sign that the appetite was returning. Such a literal interpretation of Nature's act is wholly unwarrantable. She takes away the appetite to give the affected organ or organs an opportunity to rest—of first importance in restoration to health. But there are other organs to be thought of; those which are unaffected have work to do, and they must be kept employed. The unaffected organs have even more than their own work to perform; they must do as much as possible of the work of the organs which are crippled by the disease, and so sustain or even increase the patient's vitality. Hence to withhold food entirely while the appetite is absent would be in many cases dangerous in the extreme.

In order to properly adjust the diet in any case of sickness, we must know not only what organs are to be allowed to rest, but what foods are to be excluded to enable them to do that; also what foods which will yield the most support to the patient the other organs can best dispose of, and not weaken when worked harder than usual. In chronic diseases every organ must be made to do its utmost. Food must be selected with exceeding care, and be properly disposed of by the system.

He who could properly be intrusted to feed the seriously ill must have other qualifications than these; there is yet much more for him to know. Some foods in certain diseases have really a curative effect, as, for instance, coffee. A five-ounce cup of strong coffee contains about 66 grains of extract, or an equivalent to about two grains of caffeine—often quite sufficient to relieve a neuralgia or a headache. Beef tea containing a generous quantity of red pepper is quite equal to drugs in the treatment of delirium tremens. In inflammation of the stomach and intestines, liquid food made of gelatine, isinglass, Irish moss and flaxseed, have a very soothing effect, and in some cases recovery takes place under their use alone.

And so we might go on with evidence of the curative effects of many foods. From what has been said people ought to be able to understand that the duties of the physician are by no means confined to drugging, and that when in any case he is limiting his treatment to dietetic means, he is doing what demands equally as much skill as would the proper administration of medicines. And here it is well to say that physicians of the present, whenever they can do so, employ Nature's simple dietetic remedies, always in preference to drugs. Life depends upon diet, and the restoration of health depends upon the same principles as its preservation. Disease is the result of the violation of the laws of health; hence the first step toward recovery is to establish those laws. The material for repair and support must come from diet, and often in disease a cure takes place under its proper administration alone.

There is yet one other point to bring forward in relation to feeding the seriously sick to show that the application of dietetic measures can never properly be intrusted to other than skilful hands. No two patients are alike, hence no two will scarcely demand exactly the same treatment. One may require that the nourishment be generous, in fact, all that the system can dispose of, and yet, for the other, ill with the same disease, a low diet will be much the best.

It is rarely easy, and almost always very difficult, to persuade the sick to take nourishment in sufficient quantity, and the successful nurse must be rich in expedients. Her persuasive power must be great. She must be patient, and yet firmly persistent, until her whole duty is done. There are certain general rules for her to observe. A few of them we will give. All foods for the sick should be of the very best quality, well cooked, palatably seasoned and attractively served. A savory dish will always sharpen the appetite of one in health, and it must have a stimulating influence upon a debilitated patient, to whom the flat and insipid preparations usually offered are loathsome and even nauseating.

Surprise is frequently a useful element in the dietetic treatment of the sick. Something unexpected will often be acceptable, when were the patient consulted and advised of what was being prepared for him, would take away all appetite for it. Cooking in the sickroom is, of course, always forbidden, nor should the smell of food be allowed to reach the patient if it is possible to prevent it. Absolute neatness in the service of food is a prime consideration. There is more to the patient in clean napkins, spotless china, etc., than many think. A slovenly nurse is out of place anywhere. If the doctor directs that certain foods be given hot, he means that they should be hot, and not merely warm, in which condition some are very insipid.

Occasionally one sees the nurse tasting the food in the presence of the patient—a most unpardonable habit. No more food should be at one time taken into the sickroom than is likely to be eaten, and whatever is not eaten should be at once removed. Nurses often leave it in sight, in the hope that the patient may want it a little later, but almost invariably they are disappointed. It is quite a common thing for the physician to find milk in a glass or pitcher standing near the bedside of his patient, and often the appearance of the glass is such that even a person with the strongest kind of a stomach would not care to drink from it. Of all foods, milk probably takes up impurities the easiest. Hence, to keep it exposed to the air of a sickroom or any other bad air is to simply render it unfit for use. A nurse with anything approaching neatness would never allow a glass which has held milk to be used a second time without careful washing and rinsing. Some consult their own convenience altogether too much in feeding the sick.

Food ought to be given at regular intervals when possible. If given punctually at fixed hours, a habit not only of taking, but of digesting it will soon be acquired, for our most automatic functions are influenced by custom. In this connection there is a rule which nurses should never violate. It is that of keeping for the physician an accurate record of the quantity of food taken at each feeding. As to whether or not a patient should be aroused from sleep for food depends upon the disease from which he suffers and upon his condition ; the physician, of course, must advise. As a general rule it is not best to awaken a patient for the purpose of feeding except in desperate cases. In feeding helpless patients, the food must be given slowly, and in quantities easily disposed of. See that each morsel is swallowed before another is given. A drinking tube is always of great assistance, and it is better to use that, when possible, than the spoon, not only for the reason of convenience to the nurse and comfort to the patient, but because he will often take larger quantities in this way than he can be induced to in any other. Drying the lips and the corners of the mouth after feeding is a necessary precaution against sore lips, which not infrequently result from want of this little care.

There are, doubtless, many people who do not know that it is usually quite safe to allow the fever patient suffering from great thirst all the water he wants. In fact, cold water is held as a remedial agent, a potent means of reducing fever. The effect of a glass of ice water is often immediate and marked, the fever and restlessness of the patient being noticeably lessened thereby. He who is tortured by thirst always wants a glass full of water held to his lips. Therefore, if he is to be denied all he wants, use a small glass, but at least have it full.

Bits of ice are always harmless, and are often very refreshing. Pieces the size of peas when swallowed will not infrequently relieve nausea. This suggests that there should be exercised care in the selection of ice which is to be used in the sickroom. It must be clear and clean ; snow ice is forbidden, as being the most likely to hold impurities. Here is an expedient for keeping ice for the sick : Cut a piece of clean flannel about eight inches square. Put this—after making a small hole in its centre for the water to run through—over the top of a glass tumbler; press the flannel down to half or more of the depth of the tumbler, then bind the flannel fast to the tumbler with a tape or cord, or confine it by an elastic band. When ice is put into this flannel cup, lay over it another piece of clean flannel, three or four inches square. So covered, it will keep for hours, even in warm weather.

A word about milk, the most reliable food in sickness. The best is none too good, and the best is not easily obtained in cities. As a rule, it is poor in cream, certainly it is never too rich in that important element. Where milk is to be mainly depended upon for feeding a sick person, a specimen of that furnished should be submitted to the attending physician for his examination. Oftentimes he will suggest that a quantity of cream be added to render it sufficiently nourishing. The question is often asked how much milk should a patient take in twenty-four hours to sustain him when that is his sole food. It depends, of course, upon how great a drain his system is suffering from. The minimum quantity needed is probably two quarts of milk, or three pints of milk and one pint of cream. In any case of serious illness the latter must not be given without the physician in attendance advises it. Wherever it can be given and well borne on the stomach, it will prove a most valuable form of nourishment, and it has even a tonic effect.

